# Feasibility of establishing a Canadian Obstetric Survey System (CanOSS) for severe maternal morbidity: results of a nationwide survey

**DOI:** 10.1016/j.puhip.2025.100650

**Published:** 2025-08-21

**Authors:** Isabelle Malhamé, Rebecca J. Seymour, Rizwana Ashraf, Paige Gehrke, Joseph Beyene, Tegwende Seedu, Rashid Ahmed, Susie Dzakpasu, Sara Thorne, Deshayne Fell, Amy Metcalfe, Kenneth K. Chen, Stephen Lapinsky, Leslie Skeith, Beth Murray-Davis, Josie Chundamala, Sarah A. Hutchinson, Thomas Van den Akker, Maria B. Ospina, Prakesh S. Shah, K.S. Joseph, Heather Scott, Jon Barrett, Jocelynn Cook, Marian Knight, Rohan D'Souza

**Affiliations:** a Department of Medicine, McGill University Health Centre, Montreal, Quebec, Canada; b Centre for Outcomes Research and Evaluation, Research Institute of the McGill University Health Centre, Montreal, Quebec, Canada; c Department of Obstetrics & Gynaecology, McMaster University, Hamilton, Ontario, Canada; d Department of Health Research Methods, Evidence and Impact, McMaster University, Hamilton, Ontario, Canada; e Division of Surveillance and Epidemiology, Maternal and Infant Health Section, Public Health Agency of Canada, Ottawa, Ontario, Canada; f Division of Cardiology, Pregnancy & Heart Disease Program, Mount Sinai Hospital & University Health Network, University of Toronto, Toronto, Canada; g Department of Obstetrics and Gynecology, University of Ottawa, Ottawa, Ontario, Canada; h Department of Obstetrics and Gynaecology, University of Calgary, Calgary, Alberta, Canada; i Departments of Medicine and Obstetrics & Gynecology, Women and Infants Hospital of Rhode Island, Providence, RI, United States of America; j Interdepartmental Division of Critical Care Medicine, University of Toronto, Toronto, Canada; k Department of Medicine, University of Calgary, Calgary, Alberta, Canada; l Department of Community Health Services, University of Calgary, Calgary, Alberta, Canada; m McMaster Midwifery Research Centre, McMaster University, Hamilton, Ontario, Canada; n Patient Partner, Canada; o Department of Obstetrics & Gynaecology, Leiden University Medical Centre, Leiden, the Netherlands; p Athena Institute, VU University Amsterdam, Amsterdam, the Netherlands; q Department of Public Health Sciences, Queen's University, Kingston, Ontario, Canada; r Department of Pediatrics, Mount Sinai Hospital, University of Toronto, Toronto, Ontario, Canada; s Department of Obstetrics & Gynaecology, University of British Columbia and the Children's and Women's Hospital of British Columbia, Vancouver, British Columbia, Canada; t School of Population and Public Health, University of British Columbia, Vancouver, British Columbia, Canada; u Department of Obstetrics & Gynaecology, Dalhousie University and the IWK Health Centre, Halifax, Nova Scotia, Canada; v The Society of Obstetricians and Gynaecologists of Canada, Ottawa, Ontario, Canada; w National Perinatal Epidemiology Unit, University of Oxford, United Kingdom

**Keywords:** Obstetric Survey System, Severe maternal morbidity, Feasibility study, Nationwide survey, Maternal mortality, Pregnancy-related mortality, Near-miss events, Maternal near-miss

## Abstract

**Objective:**

Obtaining data on events, processes, and circumstances leading to severe maternal morbidity (SMM) could enable targeted interventions to improve care. We aimed to assess the feasibility of gathering such data from across Canada through an Obstetric Survey System (CanOSS).

**Study design:**

A nationwide survey.

**Methods:**

We administered the electronic survey in French or English to birthing unit leads across all Canadian provinces and territories using REDCap. We presented pooled participation rates (95 % confidence intervals [CI]) across birthing units from lowest, medium, and highest tiers of service using Freeman-Tukey double arcsine transformations and common-effect models.

**Results:**

Of the 289 birthing units across Canada, 167 (57.8 %) participated in the survey. Pooled participation rates per province and territory stratified by highest, medium, and lowest tiers of service were 91.5 % (95 % CI [73.4, 100]), 58.6 % (95 % CI [48.5, 68.6]), and 54.4 % (95 % CI [41.7, 66.3]), respectively. Units reported postpartum hemorrhage (82.5 %), hypertensive disorders (65.7 %), infections (35.0 %), venous thromboembolism (16.0 %), and maternal birth injuries (15.4 %) as the leading causes of SMM. Most birthing units (80.3 %) had a system in place for reviewing SMM events. Although most review systems involved multidisciplinary expert panels with representation from birthing unit leads (82.0 %), nursing (78.0 %), and obstetrics (73.7 %), specialties, such as obstetric anaesthesia (42.4 %), midwifery (41.5 %), and internal medicine (16.9 %), were underrepresented. Lessons learned were rarely shared outside the hospital and never shared beyond regional health authorities. Importantly, 76.2 % of respondents were willing to contribute anonymized SMM data within a centralized reporting system.

**Conclusions:**

Most responding Canadian birthing units have a process in place to review SMM and would be willing to share anonymized data as part of a centralized initiative, thereby demonstrating the feasibility of leveraging existing infrastructures to establish CanOSS.

## 1 Introduction

Severe maternal morbidity (SMM) refers to a set of critical outcomes related to pregnancy, labour, childbirth, and the postpartum period that may result in severe illness, prolonged hospitalization, and long-term disability [1,2]. As maternal mortality rates have decreased and stabilized in high-income countries, such as Canada over recent decades, examining and addressing SMM has become essential to monitor and improve the quality of pregnancy-related care [3,4].

Epidemiologic studies are essential for describing trends and regional variations in SMM incidence, evaluating risk-factors, risk markers and predictors of SMM, and identifying high-priority conditions for targeted SMM reduction efforts at a national scale [5]. However, these studies often rely on datasets that provide limited information about the sequence of events leading to SMM, the interaction of clinical, social, and structural factors contributing to SMM, provider- or systems-related mechanisms contributing to SMM, and potential preventive interventions [5]. Thus, there is an urgent need to understand the root-causes and factors underlying individual SMM events to implement specific mitigation strategies and improve care. Such an approach would aim to explore the “why” behind these events, moving beyond quantitative estimates to adopt a “beyond the numbers” framework, as recommended by the World Health Organization (WHO) [6].

Obstetric Survey Systems (OSS) are centralized, anonymized, case-reporting systems for SMM events. These systems facilitate the collection of information by engaging with maternity units for conducting in-depth appraisals of individual SMM events, while respecting regional circumstances and priorities [7]. Since 2005, the United Kingdom (UK) has successfully gathered granular data on serious and rare pregnancy complications through its UK Obstetric Surveillance System (UKOSS) [8]. The success of UKOSS has prompted other countries to develop a national OSS, eventually forming the International Network of Obstetric Survey Systems (INOSS) [9].

Canada currently lacks a national OSS. As a first step, we sought to assess the feasibility of gathering detailed and qualitative SMM data from birthing units through a proposed Canadian Obstetric Survey System (CanOSS). Specifically, we aimed to: 1) map out birthing units and identify unit leads across Canada; 2) determine the presence and nature of local and/or regional systems currently in place for reviewing SMM events; and 3) assess the willingness to share anonymized data from local SMM reviews as part of the CanOSS initiative.

## 2 Methods

This descriptive, cross-sectional survey study was nested within a larger sequential mixed-methods study to assess feasibility as well as barriers and facilitators to establishing a proposed CanOSS [10]. Data were collected separately for quantitative and qualitative components of the planned study. We herein describe the quantitative component of our study, a nationwide survey, following the Checklist for Reporting Results of Internet E-Surveys (CHERRIES) Guidance [11]. Qualitative findings from interviews will be reported separately.

### 2.1 Sampling and recruitment

The study sample comprised birthing unit leads (including physicians, nurses, or administrators) from centres providing birthing services across Canada. These birthing units are part of the national public healthcare system. Unit leads were identified through a comprehensive process. First, we mapped out birthing units across the country, building on work previously conducted by members of our team [12]. Second, we used a snowball sampling approach to connect with knowledge users across provinces and territories, starting with knowledge users on our team, to ensure the completeness of this list based on their expertise within local contexts. Third, we partnered with provincial organizations to review and verify the accuracy of the birthing units list. We subsequently approached points of contacts by email or by phone for each birthing unit. Points of contact either self-identified as a unit lead or redirected the team to the most appropriate unit lead. Unit leads were then invited via email to complete the survey. In the absence of response, a first set of email reminders was sent consisting of weekly reminders for 5 weeks, and a second set of reminders was sent 3–4 months later. If the lack of response persisted, an alternative unit lead was sought. Given the large number and distribution of birthing units across the country, an in-person approach was not feasible.

### 2.2 Data collection

#### 2.2.1 Survey development

A multidisciplinary core group developed an online survey. The survey was assessed for content validity, clarity, and readability by our larger study team (including clinicians involved in SMM reviews), and refined iteratively. As part of this process, it was also tested for usability prior to study initiation. The final survey (available in French and English) comprised three main sections assessing the following components: 1) details on SMM, including the unit's commonly used term for SMM, the five most common causes of SMM, and the methods used to identify these causes; 2) the local SMM review system, such as the infrastructure and processes for reviewing SMM events and sharing results; 3) the willingness and preferred methods for sharing data with the proposed CanOSS; and 4) whether they would be willing to participate in an interview to discuss barriers, facilitators and their perspectives on establishing CanOSS. The survey incorporated both multiple-choice and open-ended questions. The online survey was distributed over 2 pages (3 questions on page 1, 18 questions on page 2, 3 questions on page 3) and comprised a minimum of 11 and a maximum of 24 questions, depending on conditional displaying of questions. The complete survey along with a description of adaptive questioning (i.e., branching logic) is available in the Supplemental Material.

#### 2.2.2 Survey administration

A unique survey link was sent to each maternity unit lead, who completed the survey on behalf of their team. The system was designed to prevent duplicate survey entries. Informed consent was sought prior to starting the survey, with a description of the purpose of the research, information on data storage and privacy, and contact information for the study team. No incentives were offered. The survey data were collected and managed using REDCap, an electronic data capture tool which was hosted at McMaster University [13]. Respondents were able to save, review, and change their answers prior to submitting each page. The survey was open to participants as of April 2022. Given a planned study end date in September 2024, survey responses were finalized in March 2024 to allow 6 months for analyses.

### 2.3 Ethical considerations

Ethics approval for this study was obtained from the Hamilton Integrated Research Ethics Board (HiREB Project #14002).

### 2.4 Analyses

Although SMM can occur at any birthing facility (or even at home), hospitals from higher tiers of service are usually optimally equipped, in terms of human and material resources, to manage these complex cases. In contrast, hospitals from medium tiers may not have the ability to provide care for all types of SMM, and hospitals from lower tiers often do not have critical care units and therefore do not have the capacity to manage certain types of SMM. As such, hospitals from lowest tiers and many hospitals from medium tiers would have to transfer patients with SMM to hospitals from higher tiers of service. To reflect the anticipated frequency and relevance of SMM reviews according to hospital type, survey response rates were stratified according to each responding unit's corresponding tiers of service. To this end, we regrouped centres into tiers of service according to level of maternal risks based on the description of individual centres' maternal and perinatal health care infrastructure, and tiers of service of equivalence were subsequently estimated at the national scale. For example, a highest level of maternal risk in Ontario (Levels III a and b; i.e., with capacity for high maternal risk and/or complex care from a medical, surgical, or obstetrical standpoint) were considered equivalent to the highest level of maternal risk in British Columbia (Tiers 5 and 6; i.e., with capacity for complicated pregnancy with medical or surgical conditions that may impact the wellbeing of the fetus). Medium level of risk usually involved capacity for low-to-moderate risk pregnancies, while lowest level of risk could accommodate low-risk pregnancies with or without capacity for caesarean birth on site, depending on units. See Table S1 of the Supplemental Appendix for a full description of the tiers of service equivalence estimates at the national scale.

Survey responses were analyzed using descriptive statistics and are reported using counts (with denominators reflecting the numbers of respondents to individual questions) and percentages. The survey participation rate was defined as the ratio of unique participants who consented to participate/unique survey links sent [11]. We obtained pooled participation rates (proportions) by synthesizing data across provinces and territories using Freeman-Tukey double arcsine transformations and common-effect models [14]. The Freeman-Tukey double arcsine transformation has several statistical advantages, including making the distribution of proportions more like a normal distribution, stabilizing variances, and avoiding the use of ad-hoc approaches to dealing with zero events. Moreover, it incorporates not only the different sample sizes across provinces and territories, but also the absolute number of responses. The survey completion rate was defined as the ratio of users who finished the survey (i.e., those who responded to the question common to all participants on the last page of the survey)/users who agreed to participate [11]. Missing data were not included in denominators. Answers to open-ended questions were analyzed using content analysis to construct qualitative summaries.

## 3 Results

### 3.1 Birthing unit characteristics

**Fig. 1:**
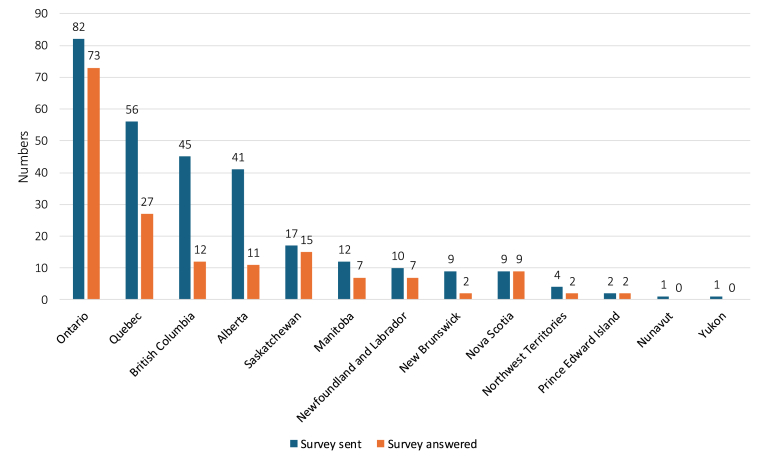
Responses per provinces and territories Legend – Lower than expected response rates in certain provinces and territories may reflect the need for more time to engage in review/surveillance activities at the hospital and regional health network levels.

**Fig. 2:**
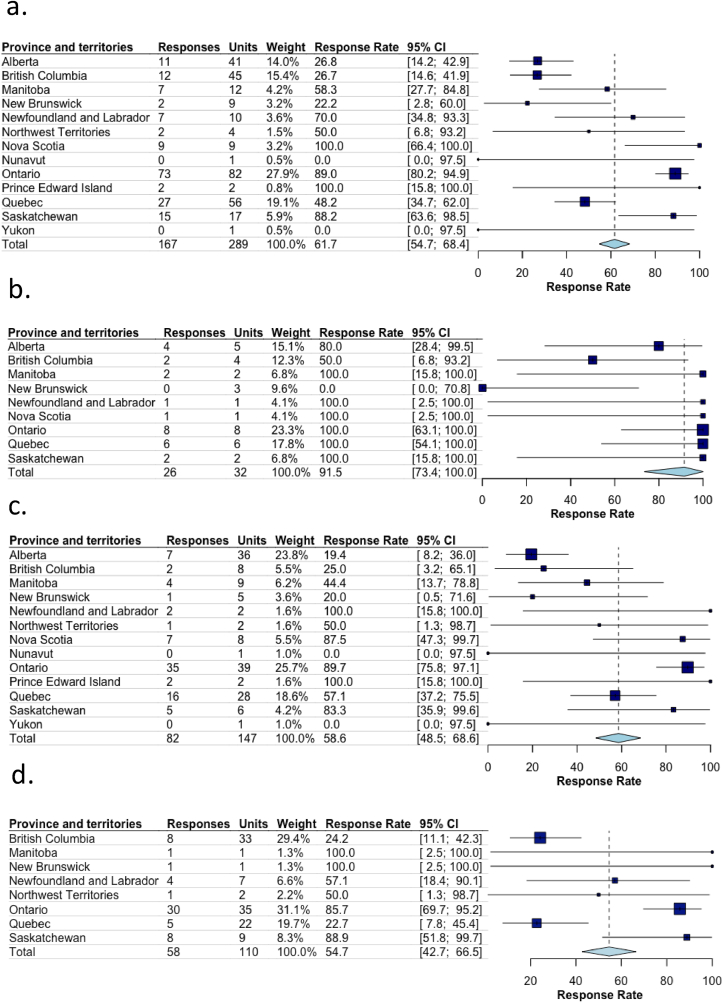
Pooled survey participation rate overall and stratified by tiers of service Legend – Pooled participation rates a. overall, b. in higher tiers of service, c. in medium tiers of service, d. in lower tiers of service. Note: based on our classification system, there were no highest level hospitals in Prince Edward Island, North West Territories, Nunavut, and Yukon. There were no low-risk units in Alberta, Nova Scotia, Nunavut, and Yukon.

In total, we identified 289 birthing units and their lead or contact person across the country. Of these, the survey participation rate was 57.8 % (n = 167/289) (Fig. 1 and Table S2 of the Supplemental Appendix). When stratified by tiers of service, the pooled participation rates (95 % CI) per provinces and territories for units in highest, medium, and lowest tiers of service were 91.5 % (95 % CI [73.4, 100]), 58.6 % (95 % CI [48.5, 68.6]), and 54.4 % (95 % CI [41.7, 66.3]), respectively (Fig. 2). The survey completion rate was of 89.8 % (n = 150/167). Most units were community hospitals (n = 104/161, 64.6 %), followed by referral care units (n = 50/161, 31.1 %), and independent or out-of-hospital care units (n = 4/161, 2.5 %).

### 3.2 Units’ descriptions of severe maternal morbidity

The terms used by units to describe SMM varied widely (Table 1); “serious adverse pregnancy outcome” and “maternal morbidity” were preferred in 34 % (n = 54/159) and 27.7 % (n = 44/159) of units, respectively, while “severe maternal morbidity” was used by 20.1 % (n = 32/159). Other terms used by 10.1 % (n = 16/159) of respondents included “critical incidents”, “reportable events”, “unexpected adverse outcomes”, “significant complications”, “sentinel events”, “unanticipated poor obstetrical/neonatal outcomes”, “unintended maternal outcomes”, and “serious untoward events”. A few participants also reported referring to the specific type of condition comprising the adverse outcomes (e.g., “amniotic fluid embolism”) instead of a general term.

**Table 1:** Understanding severe maternal morbidity at the facility level.

Item	n (%)
Term used to describe serious adverse maternal events (n = 159)	
Serious adverse pregnancy outcome	54 (34.0 %)
Maternal morbidity	44 (27.7 %)
Severe maternal morbidity (SMM)	32 (20.1 %)
Serious untoward event	7 (4.4 %)
Maternal near-miss event	6 (3.8 %)
Other^a^	16 (10.1 %)
Top five causes of SMM (n = 143)	
Postpartum hemorrhage	118 (82.5 %)
Hypertensive disorders of pregnancy	94 (65.7 %)
Sepsis/Infections	50 (35.0 %)
Venous thromboembolism	23 (16.0 %)
Maternal birth injuries	22 (15.4 %)
Other^a^	13 (9.1 %)
Identification method for unit's top causes of SMM (n = 145)	
Clinical experience	101 (69.7 %)
Internal statistics	51 (35.2 %)
External report	8 (5.5 %)
Other^a^	8 (5.5 %)

SMM = Severe Maternal Morbidity.

a Other responses are summarized in the Results section.

The five leading reported causes of SMM in birthing units (“*What are the most common types of severe maternal morbidity in your maternity unit?*” listed in no particular order) are shown in Table 1. These included postpartum hemorrhage (n = 118/143, 82.5 %), morbidity events related to hypertensive disorders of pregnancy (n = 94/143, 65.7 %), sepsis/infections (n = 50/143, 35.0 %), venous thromboembolism (n = 23/143, 16.0 %), and maternal birth injuries (n = 22/143, 15.4 %). Other causes of SMM identified (n = 13/143, 9.1 %) included mental health and addiction-related events, organ-system-specific morbidity (mostly cardiac and renal), amniotic fluid embolism, unplanned need for critical care unit admission, and anaesthetic complications. Causes unrelated to SMM, such as serious fetal events (e.g., stillbirth and cord prolapse) and maternal mortality, as well as certain conditions not traditionally considered as SMM (i.e., more commonly representing comorbidities or risk factors such as gestational or pre-existing diabetes, obesity, cholestasis, “poor prenatal history”, and a history of large or small for gestational age baby) were also reported in the “other” category. While 35.2 % (n = 51/145) of all units identified their top five causes of SMM through the use of internal statistics, most respondents (n = 101/145, 69.7 %) mentioned that the list was generated based on their experience as unit leads or SMM review system representatives. Other methods reported to generate this list included existing committees (e.g., mortality and morbidity committees or quality assurance committees), quality indicators, or reporting systems (e.g., maternal warning signs or critical incident reporting systems) informed the reporting of the top causes of SMM in their units.

### 3.3 Case review infrastructure

The majority of respondents reported that their units had a system in place for reviewing SMM (n = 118/147, 80.3 %). Of those that did not have a review system in place (n = 29/147, 19.7 %), the main reasons mentioned were the historical absence of SMM review processes (n = 12/29, 41.4 %) and a perceived low frequency of SMM due to the low-risk population cared for by the unit (n = 10/29, 34.5 %). Other reasons for lack of formal SMM reviews (n = 6/29, 20.7 %) included a lack of prioritization of reviews by leadership, reluctance of healthcare providers to participate in the absence of institutional requirement, human resource and infrastructure shortages, and presence of an informal and inconsistent case-by-case review process.

#### 3.3.1 Frequency of reviews and team compositions

The format of SMM case reviews is described in Table 2. Reviews most often occurred on an as-needed basis (n = 56/116, 48.3 %). Conversely, they took place at monthly intervals in a quarter of units (n = 28/116, 24.1 %). Other units (n = 9/116, 7.8 %) adopted hybrid approaches, for instance, as needed while leveraging existing rounds to review potential cases. Team structure and membership varied across units. Most commonly, a hospital management team (i.e., hospital appointed review team) (n = 29/118, 24.6 %) or a dedicated risk management team (n = 28/118, 23.7 %) reviewed events. Other types of teams (n = 31/118, 26.3 %) were also described, including teams reviewing all events involving maternal, fetal and neonatal morbidity and mortality, groups of volunteer physicians (obstetricians or family physicians), or interdisciplinary teams, such as leadership (operational managers, nursing management, risk management, department chief, and nursing educator) and Quality Improvement or Assurance committees.

**Table 2:** Severe maternal morbidity review infrastructure.

Item	n (%)
System in place for reviewing SMM (n = 147)	
Yes	118 (80.3 %)
No	29 (19.7 %)
If no system in place, reason described (n = 29)	
“Our unit has never formally reviewed SMM”	12 (41.4 %)
Low-risk patient population and SMM infrequent	10 (34.5 %)
Not sure why	8 (27.6 %)
Confidentiality restrictions	1 (3.4 %)
To avoid a culture of blame	1 (3.4 %)
Other^a^	6 (20.7 %)
The reviews are occurring (n = 116)	
As required/as cases arise	56 (48.3 %)
Monthly	28 (24.1 %)
Quarterly	18 (15.5 %)
Annually	5 (4.3 %)
Other^a^	9 (7.8 %)
Team reviewing SMM events (n = 118)	
Hospital management team	29 (24.6 %)
Dedicated risk-management team	28 (23.7 %)
Ad hoc committee	23 (19.5 %)
Elected sub-committee	7 (5.9 %)
Other^a^	31 (26.3 %)
Findings of review are discussed between members of the SMM review committee (n = 118)	
Yes	109 (92.4 %)
No	1 (0.8 %)
Unsure/unknown	8 (6.8 %)
The findings are shared with (n = 117)	
Healthcare professionals involved	89 (76.1 %)
Entire department/division	61 (52.1 %)
Patients and families	49 (41.9 %)
Administrative staff	31 (26.5 %)
Other^a^	22 (18.8 %)
How are findings discussed between members of the maternity team (n = 118)	
Debriefing meeting for members involved in the case	79 (66.9 %)
Open maternal morbidity and mortality meeting	65 (55.1 %)
Closed meeting of risk management team	41 (34.7 %)
Not discussed	1 (0.8 %)
Other	15 (12.7 %)
A written report is prepared following the meeting (n = 116)	
Yes	87 (75.0 %)
No	14 (12.1 %)
Sometimes	15 (12.9 %)
Lessons learned are formulated into recommendations and incorporated into local protocols/policies/guidelines (n = 117)	
Yes	91 (77.8 %)
Sometimes	25 (21.4 %)
No	1 (0.9 %)
Unit conduct audits to ensure that the recommendations are being implemented and result in clinical improvements (n = 118)	
Yes	61 (51.7 %)
No	57 (48.3 %)
Audit's frequency (n = 118)	
As needed	30 (49.2 %)
Quarterly	13 (21.3 %)
Annually	7 (11.5 %)
Monthly	5 (8.2 %)
Other^a^	6 (9.8 %)

SMM = Severe Maternal Morbidity.

a Other responses are summarized in the Results section.

**Fig. 3:**
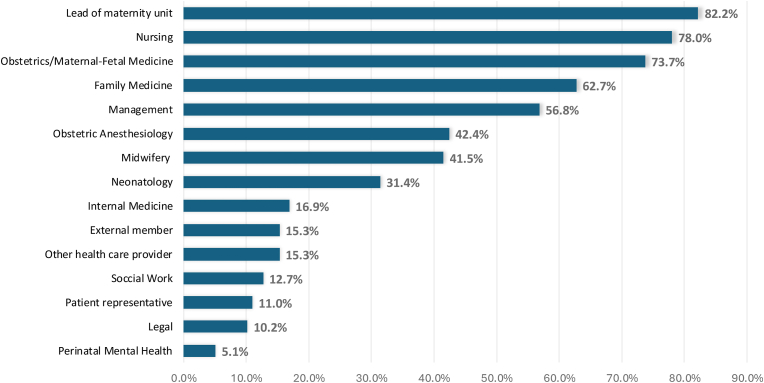
Team members involved in severe maternal morbidity reviews ∗Internal Medicine (including Obstetric Medicine, Internal Medicine, Critical Care).

The teams varied in terms of composition of disciplines involved and the context in which they would be involved (Fig. 3). While some stakeholders were represented in 50 % or more of the review teams (e.g., leadership of the maternity unit [82.0 %], Nursing [78.0 %], Obstetrics/Maternal-Fetal Medicine [73.7 %], Family Medicine [62.7 %], and Management [56.8 %]), others were included in only a minority of teams (e.g., Obstetric Anaesthesia [42.4 %], Midwifery [41.5 %], Neonatology [31.4 %], Internal Medicine (Obstetric Medicine/Internal Medicine/Critical Care) [16.9 %], and other health care providers [15.3 %]) (Fig. 3).

#### 3.3.2 Sharing of findings and recommendations

The processes for sharing findings and recommendations of reviews is described in Table 2. Respondents reported that findings were discussed among members of the review committee in almost all instances (n = 109/118, 92.4 %), whereas in 9/118 instances (7.6 %) respondents were either not certain or stated that review committee members did not discuss findings among themselves. In addition, upon completion of the review, 76.1 % (n = 89/117) of facilities reported sharing the review findings with healthcare professionals involved in the event, while 52.1 % (n = 61/117) reported sharing the findings with members of the clinical department or division. Only 41.9 % (n = 49/117) reported sharing findings with the patient or family and 26.5 % (n = 31/117) with hospital/facility administration. Some participants (n = 22/117, 18.8 %) reported discussing findings at the level of the zonal/regional health authorities, while others were uncertain whether findings were shared, and if so, with whom. No facility reported sharing data beyond the regional health authorities. When findings were not universally shared outside the review committee, the target audience depended on a variety of factors, including the type of event, the level of review conducted, the outcomes of the review, and the stakeholders involved. Where review findings were shared, several forums were used for dissemination, including debriefing sessions for those involved (n = 79/118, 66.9 %), open (departmental) maternal morbidity and mortality meetings (n = 65/118, 55.1 %), and closed meetings with a smaller group (n = 41/118, 34.7 %). Following a review, a written report was prepared in 75 % (n = 87/116) of cases, while among those sometimes preparing a written report (n = 15/116, 12.9 %), findings could also be disseminated through education, newsletters, rounds, or on bulletin boards.

Lessons learned were formulated into recommendations for local practice change in 77.8 % (n = 91/117) of instances. Among respondents where lessons were only sometimes formulated into recommendations (n = 25/117, 21.4 %), some reported that the decision to formulate recommendations was made on a case-by-case basis and influenced by the presence of existing guidelines that already included the recommendation, and the review committee's perception on whether recommendations were warranted (indicators for what would warrant a case review were not described). Audits to ensure implementation of recommendations were carried out in 51.7 % (n = 61/118) of units, most commonly on an as-needed basis (49.2 %) or quarterly (21.3 %). Others (n = 6/118, 9.8 %) reported that the frequency of audits depended on the suggested recommendations and the resources available to support the audit. It was also described that, while audits were conducted to ensure recommendations are implemented, changes in outcomes were not necessarily evaluated.

### 3.4 Willingness to share and preferred method for sharing

The majority (n = 112/147, 76.2 %) of participants expressed willingness to share anonymized data on SMM occurring in their unit to a centralized survey system (while considering that privacy and research ethics concerns had been addressed). Although 22.4 % (n = 33/147) of respondents reported being unsure, only 1.4 % (n = 2/147) were not willing to share data. The preferred method for data sharing was through a dedicated web-based entry platform (n = 94/145, 64.8 %), followed by: a standardized written report describing the SMM event, filled out by the team and sent to a centralized destination (n = 18/145, 12.4 %); anonymized data from medical record and sent to centralized destination (n = 14/145, 9.7 %); and a standardized slide deck for describing a SMM event filled out by their team and sent to a centralized destination (n = 2/145, 1.4 %). Some respondents suggested that leveraging existing regional or provincial electronic systems that already collect SMM-related data would be an effective method to send anonymized data. A few noted uncertainty about what would work best, or if it would be supported by their hospital, while others estimated that dedicated staffing or funding would be required. Following the online survey, a total of 77 respondents agreed to participate in a subsequent qualitative interview.

## 4 Discussion

This paper describes the findings of a nationwide survey to evaluate the feasibility of establishing a Canadian Obstetric Survey System (CanOSS). By mapping out all birthing units in Canada, identifying unit leads, and capturing responses from a representative sample of birthing units, we have described the local understanding of SMM, variations in existing infrastructure and processes for reviewing SMM events, and the willingness to share anonymized data in a centralized reporting system, thereby demonstrating the feasibility of leveraging existing infrastructures to establish CanOSS.

Although a clear and operationalized definition for the term “severe maternal morbidity” is routinely used for epidemiologic surveillance in Canada [15,16], this term does not appear to be uniformly adopted in clinical practice. In fact, we observed the use of a variety of alternate terms to describe SMM, with most units referring to “serious adverse pregnancy outcomes”. This may reflect the varying complexity of pregnancy complications managed at different tiers of service, with lower tier centres reviewing complications that do not necessarily concur with operationalized definitions of SMM. Therefore, for purposes of conducting national surveillance through CanOSS, it is essential to ensure a common understanding of conditions requiring review.

In keeping with epidemiologic studies on SMM trends in Canada [15,16], respondents confirmed that the five leading causes of SMM were related to events usually considered to be direct (obstetric) causes and included postpartum hemorrhage, hypertensive disorders of pregnancy, sepsis/infections, venous thromboembolism, and maternal birthing injuries. Leading causes of SMM identified through this survey contrast with those reported by several high-income countries that have successfully reduced mortality and morbidity from obstetric causes through the establishment of national surveillance systems, and are now reporting indirect (non-obstetric) conditions, such as cardiovascular disease, as the leading causes of maternal morbidity and mortality [17,18]. These identified leading causes of SMM, aligning with epidemiologic data, illustrate that reducing morbidity and mortality from preventable obstetric causes should become a high priority to improve the health of pregnant women and gender diverse people.

While this study identified that most birthing units had a system in place for reviewing SMM, there was considerable variation in infrastructure, process, team composition and sharing of findings. In instances where the lack of a review system was identified, our initial contact through the feasibility survey could lead to further engagement to build and enhance capacity to conduct local reviews or contribute to regional SMM reviews. For instance, we have initiated the co-development of resources to support the implementation of high-quality facility-based SMM reviews that may be used by these units in the future. Most SMM reviews involved a multidisciplinary dedicated team, aligning with best practice guidance recommending that facility-based SMM reviews be performed by multidisciplinary review committees within a safe review culture [1,[19], [20], [21], [22]]. Respondents reported that most SMM review teams comprised a core of knowledge users directly involved in pregnancy care, whose composition reflected disciplines caring for direct causes of SMM under review. However, several disciplines involved in the care of women and gender diverse people experiencing SMM such as internal/obstetric medicine, critical care medicine, and obstetric anaesthesia were not frequently represented in the review teams. Proportionally relevant representation of diverse disciplines within SMM reviews allows for a broad range of perspectives to be included when formulating recommendations on how to improve care. As such, a multidisciplinary approach is critical to enrich proposed recommendations and ultimately to improve patient care.

Most reviews led to recommendations for local practice change, indicating that their outputs have the potential to make meaningful improvements to clinical care. While it was encouraging to note that such findings and recommendations were frequently shared with healthcare professionals involved in the event, in many instances information was not shared with other members of the department or other disciplines involved in patient care. Further, findings were reported as almost never shared with the larger clinical community or general public. In contrast, over the past 15 years, UKOSS has gathered data on over 60 serious pregnancy-related complications, and by sharing findings and recommendations nationally, have influenced clinical practice and policy, while preventing similar events from occurring at other facilities [[23], [24], [25]]. While there is room for improvement, the sharing of findings with patients and families in approximately half of all facilities is commendable, and is in contrast with reports from the literature, where review systems seldom described their communication process with families [26].

Most importantly, despite potential barriers to data sharing, such as concerns related to patient and provider confidentiality and the legal implications of sharing information on rare pregnancy-associated events [27], an overwhelming majority of respondents expressed willingness to share anonymized data on their SMM reviews, preferably using an online platform, if measures to protect confidentiality and privacy were in place. Given that most birthing units in the country have some form of SMM review infrastructure already in place (i.e., an SMM review culture, review committees, a process for generating written reports and clinical recommendations, and ongoing audits), and a willingness to share information, it is feasible to suggest the establishment of a centralized CanOSS that integrates existing infrastructures in order to report, centrally review events, share lessons learned, and formulate clinical recommendations for quality improvement at the national level.

This study had several strengths. We mapped out and contacted each maternity unit across the country to capture a comprehensive sample of survey respondents to ensure a national scope. Such groundwork is the foundation for a community of practice and network supporting the establishment of a future CanOSS. This survey also contributed to raising awareness about the need for standardized centralized reporting practices to improve care beyond individual units. The survey collected novel data that will inform research priorities, and the development of knowledge mobilization tools designed to support clinical teams conducting SMM reviews.

Limitations of this study included the fact that respondents may have represented a subset of units most likely to have structures in place for reviewing SMM and to be able to contribute to a centralized CanOSS. Nevertheless, the survey highlighted the need for continued engagement with units that did not respond and who may also benefit from SMM reviews. While hospital review committee leads were invited from across all Canadian provinces and territories to participate in the study, there were lower than expected response rates in some provinces and territories. This may reflect the need for more time for healthcare providers at hospitals and regional health networks levels to engage in review/surveillance activities. Moreover, while we captured responses from nearly all high-acuity care centres across the country, birthing units from lower and intermediate tiers of service may have been underrepresented. Results may therefore not be generalizable to care centres with lower acuity and less exposure to SMM. Given that continuity of care is critical to the quality of delivery and the overall experience of pregnancy care across settings [28], developing strategies to ensure inclusivity of lower and intermediate tiers of service in the SMM review process and centralized data collection will be of paramount importance. Such strategies may include the development of context-relevant resources to support local SMM reviews, outreach to local provincial professional organizations to provide ongoing support for centralized reporting to a future CanOSS, and continued engagement and bilateral exchanges with these units.

## 5 Conclusion

In summary, we conducted a nationwide survey to inform the feasibility of developing CanOSS to gather granular data on current approaches and identify processes to reduce the incidence of SMM going forward. The top five causes of SMM reported by individual units reaffirmed the need to continue to address obstetric drivers of SMM and mortality, in addition to non-obstetrical causes of these events. Most units in Canada have existing infrastructures to review SMM and expressed a willingness to share findings and recommendations. This indicates that existing systems could be integrated in a national reporting process to ensure that lessons learned are shared across units at the national level, thereby demonstrating the feasibility of developing a CanOSS to review SMM with an aim to reduce its incidence across Canada.

## What this study adds up

• Identifies postpartum hemorrhage, hypertensive disorders, infections, venous thromboembolism, and maternal birth injuries as the leading causes of severe maternal morbidity among surveyed birthing units in Canada.
• Demonstrates that while many birthing units in Canada have systems in place for reviewing severe maternal morbidity (SMM), there is significant variation in how these systems are implemented across the country.
• Highlights the feasibility of establishing a Canada-wide obstetric survey system to systematically review SMM - the first step in developing targeted interventions to reduce their rising rates in Canada.

## Implications for policy and practice

• The study calls for the development of a nationwide system to review and address SMM, which could improve coordination and consistency across Canada's healthcare units.
• Encourages policy makers to prioritize the sharing of local lessons learned and best practices at a national level to reduce the incidence of severe maternal morbidity.
• This study sets the stage for other countries in the Americas and beyond, to conduct similar studies with a view to reducing severe adverse events in pregnancy.

## Ethical approval

Ethics approval for this study was obtained from the Hamilton Integrated Research Ethics Board (HiREB Project #14002).

## Funding

This research was funded through a grant awarded by the 10.13039/501100000024Canadian Institutes of Health Research (CIHR) - #202104PJT-462756. The study sponsor had no role in the study design, data collection, analysis, and interpretation, in the writing of the report, and in the decision to submit the paper for publication.

## Declaration of Interest Statement

The authors declare that they have no known competing financial interests or personal relationships that could have appeared to influence the work reported in this paper.
