# Supplementary material for: Feasibility of establishing a Canadian Obstetric Survey System (CanOSS) for severe maternal morbidity: results of a nationwide survey
Source: Public Health Pract (Oxf). 2025 Aug 21;10:100650. doi: 10.1016/j.puhip.2025.100650 (PMC12445713; doi:10.1016/j.puhip.2025.100650)
Supplement: Multimedia component 1 [file mmc1.docx]

**Table S1. Tiers of Care equivalence at the national scale**

| **Level of maternal risk** | **Ontario** | **British Columbia** | **Alberta** | **Quebec** | **Saskatchewan** | **Manitoba** | **New Brunswick** | **Nova Scotia** | **Prince Edwards Island** |
| --- | --- | --- | --- | --- | --- | --- | --- | --- | --- |
| Highest | IIIb  IIIa | Tier 6  Tier 5 | Level D | Level III | Tier 4 | Level 3 | Level 3 | Tier 3 |  |
| Medium | IIc  IIb  IIa | Tier 4  Tier 3 | Level C  Level B | Level IIb  Level IIa  Level Ib | Tier 2  Tier 2a  Tier 2b | Level 2 | Level 2B  Level 2A | Tier 2b  Tier 2a | Tier 2b  Tier 2a |
| Lowest | Ib  Ia | Tier 2  Tier 1 | Level A | Level Ia | Tier 1a  Tier 1b | Level 1 | Level 1 |  |  |

**Newfoundland:**

Tiers based on hospital volume

**Northwest territories:**

Levels 1 and 2 without description

**Table S2. Response rates by provinces/territories according to Tiers of Service**

|  | **Total** | | | **Lowest risk** | | | **Medium Risk** | | | **Highest risk** | | |
| --- | --- | --- | --- | --- | --- | --- | --- | --- | --- | --- | --- | --- |
| **Provinces and Territories** | **Units** | **n** | **%** | **Units** | **n** | **%** | **Units** | **n** | **%** | **Units** | **n** | **%** |
| **Overall** | 289 | 167 | 58 | 111 | 58 | 52 | 147 | 82 | 56 | 32 | 26 | 81 |
| **Alberta** | 41 | 11 | 27 | 0 | 0 | 0 | 36 | 7 | 19 | 5 | 4 | 80 |
| **British Columbia** | 45 | 12 | 27 | 33 | 8 | 24 | 8 | 2 | 25 | 4 | 2 | 50 |
| **Manitoba** | 12 | 7 | 58 | 1 | 1 | 100 | 9 | 4 | 44 | 2 | 2 | 100 |
| **New Brunswick** | 9 | 2 | 22 | 1 | 1 | 100 | 5 | 1 | 20 | 3 | 0 | 0 |
| **Newfoundland and Labrador** | 10 | 7 | 70 | 7 | 4 | 57 | 2 | 2 | 100 | 1 | 1 | 100 |
| **Northwest Territories** | 4 | 2 | 50 | 2 | 1 | 50 | 2 | 1 | 50 | 0 | 0 | - |
| **Nova Scotia** | 9 | 9 | 100 | 0 | 0 | - | 8 | 7 | 88 | 1 | 1 | 100 |
| **Nunavut** | 1 | 0 | 0 | 0 | 0 | 0 | 1 | 0 | 0 | 0 | 0 | - |
| **Ontario** | 82 | 73 | 89 | 35 | 30 | 86 | 39 | 35 | 90 | 8 | 8 | 100 |
| **Prince Edward Island** | 2 | 2 | 100 | 0 | 0 | - | 2 | 2 | 100 | 0 | 0 | - |
| **Quebec** | 56 | 27 | 48 | 22 | 5 | 23 | 28 | 16 | 57 | 6 | 6 | 100 |
| **Saskatchewan** | 17 | 15 | 88 | 9 | 8 | 89 | 6 | 5 | 83 | 2 | 2 | 100 |
| **Yukon** | 1 | 0 | 0 | 0 | 0 | - | 1 | 0 | 0 | 0 | 0 | - |

Legend – Lower than expected response rates in certain provinces and territories may reflect the need for staff to have more time to engage in review/surveillance activities at hospitals and regional health networks levels
