# Supplementary material for: Feasibility of establishing a Canadian Obstetric Survey System (CanOSS) for severe maternal morbidity: results of a nationwide survey
Source: Public Health Pract (Oxf). 2025 Aug 21;10:100650. doi: 10.1016/j.puhip.2025.100650 (PMC12445713; doi:10.1016/j.puhip.2025.100650)
Supplement: Multimedia component 2 [file mmc2.docx]

Improving Maternal Outcomes by Engaging Stakeholders (First Page)

You are being invited to take part in a research study focused on identifying the leading causes of severe maternal morbidity in Canadian provinces. You are being asked to participate because we have identified you as the maternity unit lead/ representative for reviewing severe maternal morbidity. Your agreement to participate in this study is optional and entirely up to you. By participating in this study you do not waive any rights you may have under the law.

In order to decide whether or not you want to be part of this research study, you should understand what is involved and the potential risks and benefits. This form gives detailed information about the research study, which will be discussed with you. Once you understand the study, you will be asked to click “I agree” if you want to participate.

Please take as much time as you need to make your decision. If you would like more details about something mentioned here, or in the discussion, please ask. You should feel free to discuss this study and your participation with your family and friends.

Why is this research being done?

Canada does not have a national Obstetric Surveillance System (OSS) for gathering granular data on severe maternal morbidity. Prior to developing a nationwide OSS, it is important to determine the feasibility of gathering granular data on severe maternal morbidity from maternity units and identify challenges posed by disparate systems of maternity care delivery across and within Canadian provinces. Our study focuses on maternal care in all Canadian provinces that represent the diverse health care environments in Canada.

What is the purpose of this study?

The purpose of this study is to determine if our method of collecting severe maternal morbidity data is feasible. How many people will be enrolled in the study?

We will disseminate the survey to all the maternity units heads widely throughout Canada and our aim is to get as many participants as possible.

How is this study designed?

You are being invited to take part in a web-based survey. The survey has to be completed on REDCap Surveys using your unique link. The survey includes questions about leading causes of severe maternal morbidity in your unit, systems that have been put in place to review these events, and your ability/ barriers to sharing data with a central repository.

What will my responsibilities be if I take part in this study?

If you volunteer to participate in this study, we will ask you to complete the web-based survey. The survey is expected to take between 10-15 minutes of your time. There is also an interview portion of this study, and if interested, you can provide your name and email at the end of the survey.

What are the possible risks of participating?

This study will use the RedCap platform to collect data, which is an externally hosted cloud-based service. A link to their privacy policy is available here (https://projectredcap.org/software/mobile-app/privacypolicy/). While the Hamilton Integrated Research Ethics Board has approved using these platforms to collect data for this study, there is a small risk of a privacy breach for data collected on external servers. If you are concerned about this, we would be happy to make alternative arrangements for you to participate, perhaps via telephone. Please talk to the researcher if you have any concerns.

What are the possible benefits for me and/or for society?

We cannot promise any personal benefits to you from your participation in this study. There is no direct benefit to you for participating in this study but it will help us learn about leading causes of severe maternal morbidity, variations between regions and provinces, as well as social and clinical factors responsible for these events, which will enable the prioritization of preventative strategies.

If I do not want to participate in this study, are there other choices?

It is important for you to know that you can choose not to take part in the study. Will I be paid to participate in this study?

You will not be paid to participate in this study.

Will there be any costs?

Your participation in this research study will not involve any additional costs to you. Can participation in the study end early?

If you volunteer to be in this study, you may withdraw at any time during the study. You may refuse to answer any questions you don’t want to answer and still remain in the study.

What information will be kept private?

Your data will only be shared among members of the research team. It will not be shared with anyone else except with your consent or as required by law. All personal information such as your name and email address will be removed from the data and will be replaced with a number. A list linking the number with your name will be kept in a secure place, separate from your file. We will be collecting your name and email addresses. The study data will be securely stored in a locked office in the research office and on a secure server. The data for this research study will be retained for 10 years. The investigators agree not to attempt to connect your information back to you after the study is completed.

For the purposes of ensuring the proper monitoring of the research study it is possible that a member of the Hamilton Integrated Research Ethics Board may consult your research data. By agreeing below, you authorize such access.

If the results of the study are published, your name will not be used and no information that discloses your identity will be released or published without your specific consent to the disclosure.

It is important to note that participation in this research study will in no way affect your professional standing. If I have any questions or problems, whom can I call?

Members of the study research team are available to answer any questions you may have regarding the study. If you have questions regarding this research study, you may contact Dr. Rizwana Ashraf by calling 1-365-889-6703 or by email: [ashrar9@mcmaster.ca](about:blank)

If you have questions regarding your rights as a research participant, you may contact the Office of the Chair of the Hamilton Integrated Research Ethics Board at 905 521-2100 ext. 42013.

PARTICIPANT CONSENT [If response is “No”, survey won’t proceed] Yes

No


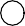

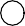


I have read the preceding information thoroughly. I agree to participate in this study.

PARTICIPANT CONSENT Yes

No


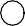

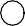


I agree to be contacted for participation in future research studies

**Survey Questions**

**PART-I**

1. The pregnancy unit I am answering this survey about a. A referral centre/ hospital is: b. A community hospital

c. An independent/ free-standing/ out-of-hospital Birthing Unit/ Maison Naissance


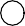

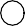

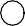


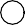
d. Other

Other (Please describe)

*Page 3*

1. To me, the term 'severe maternal morbidity' means (Please be as descriptive as possible)^1^
2. What term does your unit use to describe serious a. Severe maternal morbidity adverse maternal events occurring during pregnancy, b. Maternal morbidity

labour, childbirth or the postpartum period? c. Maternal near-miss


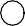

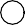

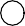

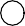

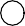

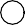


1. Serious untoward event
2. Serious adverse pregnancy outcome
3. Other - please specify

Other (Please specify)

*^1^Survey responses to this question will be reported separately*

**For the remainder of this survey, we will use the term “severe maternal morbidity" to describe**

**the events described on previous page [From Here New/Second page starts and cannot go back]**

1. What are the most common types of severe maternal morbidity (SMM) in your maternity unit? [List up to

five]

1. How did you identify your unit's top causes of
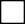
 a. Unit/internal statistics

severe maternal morbidity (SMM)?
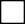
 b. Clinical Experience

c. External report

d. Other

Others (Please describe):

**Survey Questions [ Second part of the new page]**

**PART-II**

1. Is there a system in place for reviewing cases of Yes


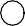

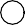


SMM occurring in your unit? No

If the answer is Yes, survey will go to question 7 onwards but skip 17. If “No”,

it will go to question “17”

1. Who reviews the cases of SMM in your unit?
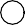
 a. Dedicated risk-management team appointed by

maternity unit

1. Hospital risk management team


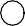

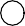

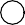


1. Elected sub-committee
2. Ad hoc committee appointed on a case-by-case basis
3.
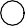
Other

Other (Please specify):

1. Which of the following disciplines are represented
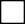
 a. Lead of Maternity Unit/ Labour and Delivery/ in the team? (Check all that apply) Department [State role please in space below]
2. Internal Medicine (Obstetric Medicine/ Internal Medicine/ Critical care physicians)
3. Obstetrician/ Maternal-Fetal Medicine Physician
4. Obstetric Anesthesiologists
5. Nurse
6. Midwife
7. Family Physician
8. Neonatologist
9. Management (please describe in space below)
10. Legal
11. Social Worker/ Chaplain
12. Perinatal Mental Health/ Wellness representative
13. Other health care providers (please describe in space below)
14. Members external to the Maternity Unit (please describe in space below)
15. Patient representative
16. Others not listed above (please describe in space below)

Please describe:

1. Are the findings of the review discussed between a. Yes

members? b. No


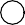

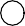

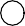


c. Unsure/unknown

1. How are the findings of the review discussed
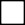
 a. Maternal Mortality and Morbidity (M&M) meeting between members of the maternity team? involving presentation of cases, open to all

members of the unit/ department

1. Closed meeting of members of the risk-management or similar team
2. Debriefing meeting of members involved in the case
3. Not discussed
4. Other


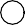

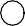

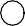


Other (Please specify):

1. How frequently are these cases reviewed? a. Monthly
2. Quarterly


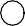

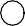

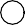

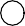

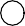


1. Annual
2. As Required/ As cases arise
3. Other

Other (Please specify):

1. Is a written report prepared following the a. Yes

meeting? b. No


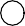

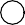

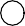


c. Sometimes

Sometimes (Please specify):

1. The findings of the review are shared with:
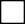
 a. Patient and family

(Check all that apply)
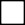
 b. Healthcare professionals involved in the event

- 1. Entire department/division
  2. Administrative staff
  3. Other

Others (Please describe):

1. Are the lessons learned, formulated into a. Yes recommendations and incorporated into local protocols/ b. No policies/ guidelines? c. Sometimes

Sometimes (Please specify):

1. Does your unit conduct audits to ensure that the Yes recommendations are being implemented and that this is No resulting in improvements in outcomes?
2. How often do audits occur? a. Monthly
3. Quarterly


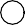

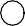

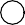

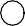

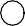

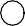

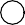


1. Annual
2. As Required/ As cases arise
3. Other

Other (Please specify):

1. Why do you think there is no system in place for
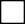
 a. Our patients are low-risk patients and SMM is review of SMM events? (Check as many as apply) infrequent
2. Because of confidentiality restrictions
3. Our unit has never formally reviewed SMM
4. To avoid a culture of blame
5. Not sure why
6. Other

Other (Please describe with as much detail as possible)

**Survey Questions PART-III**

**Countries such as the United Kingdom, Australia, and the Netherlands have developed national survey systems where National data on SMM are gathered centrally in order to identify regional variations as well as patient-, provider- and systems-specific issues that may explain the SMM. These processes and the data enable the development of targeted policies**

**and allocation of resources to improve maternal health across all communities.**

1. If a centralized surveillance system were to be a. Yes developed in Canada and research ethics approval were b. No


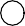

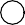

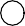


to be obtained, would you be willing to share c. Unsure anonymized data on SMM occurring in your unit?

If Yes, survey will go to question 19 and skip 20, If “No”, will go to 20

1. Which of the following methods of sending
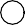
 a. A dedicated person from your unit would enter anonymized data would work better for your unit? data on all cases of SMM that occur in your unit,

into a web-based data entry platform [The frequency of data entry could be monthly, quarterly, annually or as a case occurs, as determined by you]

1.
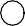
You would anonymize data from electronic medical records and paper charts and fax/send these over to a centralized destination for data extraction
2.
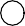
A standardized PowerPoint presentation for describing SMM events would be prepared by your team and sent to a centralized destination for data extraction.
3.
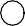
A standardized written report for describing SMM events would be filled out by your team and this written report would be sent to a centralized destination for data extraction.
4.
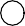
Other

Other (please describe your ideal method):

1. What are the reasons/ barriers to sharing data on
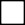
 a. Concern about patient confidentiality SMM with a centralized survey system (check all that
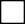
 b. Concern about blame

apply)^1^
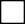
 c. Concern about time commitment

1. Unlikely to get approval from members of the unit
2. We would need more information prior to deciding
3. Lack of personnel to commit to this on an ongoing basis
4. Lack of other resources (please describe)
5. Legal/ Legislative concerns
6. Other

Other (Please describe):

20a. Can you think of any reasons other units may not

want to or would be hesitant to respond to this survey?

1. Are there any additional comments or thoughts you would like to make at this time?

*^1^Question added with an amendment in April 2023*

**Survey Questions**

**PART-IV**

1. Would you be willing to participate in a brief Yes


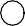

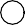


telephone call/virtual meeting to discuss a national

If Yes will go to 23, If “No” will go to End of Survey No SMM surveillance system? Topics we could discuss

include, but are not restricted to, the nature of the proposed surveillance system and barriers/challenges to gathering information on SMM specific to your unit.

1. Thank you for your willingness to talk about this

important topic. Please enter your email address so

that we can contact you to find a time that works best

for you

1. Please indicate whether you would prefer to be
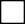
 a. French

interviewed in French or English
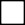
 b. English

**END OF SURVEY (Third page/form)**

1) Thank you for completing this survey. If you would

like us to send you the results of the survey, please

enter your email here
